# Expression of auxin carrier genes during adventitious rooting in *Eucalyptus globulus*

**DOI:** 10.1186/1753-6561-5-S7-P64

**Published:** 2011-09-13

**Authors:** Arthur Fett-Neto, Marcia De Almeida, Carolina Ruedell

**Affiliations:** 1 Gabriela Pereira Center for Biotechnology, Federal University of Rio Grande do Sul, Porto Alegre, RS, Brazil

## Background

*Eucalyptus globulus* and its hybrids are important for the cellulose and paper industry, mainly due to their relatively low lignin content. However, rooting of cuttings of this species is often recalcitrant and exogenous auxin application is necessary for adventitious root development. Auxin plays a central role in rooting capacity, which is particularly affected by its endogenous content and transport rate. The shoot apex is a major source of endogenous auxin, which is mainly transported by both influx (*AUX1*) and efflux (*PIN*) carriers in a specific basipetal active transport through the vascular parenchyma in stems. As part of a larger study to investigate the causes of low rooting in *E. globulus* microcuttings without exogenous auxin, we evaluated the expression profiles of *AUX1* and*PIN1* during the process of adventitious rooting using qPCR.

## Material and methods

*E. globulus in vitro* tip microcuttings obtained from 14 week-old seedlings were submitted to a culture system consisting of a two-step protocol: an initial step of induction, which lasted 96 h (induction medium composition: 0.3x MS salt concentration, 0.4 mg l^-1^ thiamine HCl, 100 mg l^-1^ inositol, zero (control) or 10 mg l^-1^ indolyl-butyric acid (IBA – root promoting auxin), equivalent to 49.3 μM, 30 g l^-1^ sucrose and 6 g l^-1^ agar, followed by a formation step (same composition of induction medium, but devoid of auxin and supplemented with 1 g l^-1^ activated charcoal). The expression analysis of the selected genes was monitored along the rooting process and the harvest of microcuttings for RNA extraction was done at 6, 12, 24, 48 and 96 h of exposure to induction medium and 24 and 48 h after transfer to formation medium (formation step), for both treatments (with and without auxin in the first step). For the formation step harvest, the microcuttings remained for 96 h in the induction medium before transfer to formation medium. Total RNA was extracted, and the first strand cDNA synthesis was performed for all of the samples starting from about 500 ng total RNA, using oligo-dT primers and reverse transcriptase M-MLV (Invitrogen) in a final volume of 20 μl. The final cDNA products were diluted 50-fold in RNAse-free distilled water prior to use in qPCR. The analysis was carried out using specific primers for *Arabidopsis thaliana* orthologue genes in eucalypt and both *Histone H2B* (*H2B*) and *Alpha-Tubulin* (*TUA*) genes were used as references [1]. The data were analysed with the comparative Ct method [2].

## Results and conclusion

The gene encoding the auxin influx carrier (*AUX1*) did not show differences in expression profile between treatments (with and without exogenous auxin), suggesting that *AUX1* is not critical to the process of adventitious rooting promoted by exogenous auxins in microcuttings. This would be in line with the fact that the rate of endogenous auxin transport is probably not limiting under exogenous auxin supply. The auxin efflux carrier gene encoding *PIN1* showed an expression increase during the first 24 hours of the induction step in microcuttings exposed to exogenous auxin when compared with the control treatment (without exogenous auxin supply). This result seems to indicate a requirement of *PIN1* to redistribute and perhaps concentrate auxin, possibly IBA-derived IAA (indolyl-3-acetic acid), in specific areas of the base of microcuttings, in order to allow root development. Although IBA is a natural auxin recognized as an IAA precursor, its use as exogenous auxin instead of IAA, a common practice in clonal propagation by cuttings, may have also involved the expression of other sets of recently described IBA-specific transporters [3]. In conclusion, despite the unusual exogenous auxin entrance pathway into the microcutting compared to the endogenous auxin fluxes, *PIN1* likely takes part in the process of auxin concentration required to program founder cells involved in the establishment of new root meristems.
